# [Retracted] Activated protein C overexpression suppresses the pyroptosis of subarachnoid hemorrhage model cells by regulating the NLRP3 inflammasome pathway

**DOI:** 10.3892/etm.2026.13265

**Published:** 2026-08-10

**Authors:** Ai Yan, Xuyan Pan, Xianqiang Wen, Xiaohu Nie, Yuntao Li

Exp Ther Med 22:1391, 2021; DOI: 10.3892/etm.2021.10827

Following the publication of the above paper, it was drawn to the Editor’s attention by a concerned reader that certain of the western blot data featured in Figs. 1B, 3C and 4C had either already been submitted for publication, or were already published, in several articles in different journals that were written by different authors at different research institutes. A subsequent investigation of this paper undertaken by the Editorial Office confirmed the authenticity of these findings.

Owing to the fact that the contentious data in the above article had either already been submitted for publication, or were already published, prior to its submission to *Experimental* and *Therapeutic Medicine,* the Editor has decided that this paper should be retracted from the Journal. Upon contacting the authors, they accepted the decision to retract the paper. The Editor apologizes to the readership for any inconvenience caused.

